# Modeling the binding of diverse ligands within the Ah receptor ligand binding domain

**DOI:** 10.1038/s41598-019-47138-z

**Published:** 2019-07-23

**Authors:** Sara Giani Tagliabue, Samantha C. Faber, Stefano Motta, Michael S. Denison, Laura Bonati

**Affiliations:** 1 Department of Earth and Environmental Sciences, University of Milano-Bicocca, Milan, Italy; 2 Curriculum in Toxicology and Environmental Medicine, University of North Carolina at Chapel Hill, Chapel Hill, North Carolina, USA; 3 Department of Environmental Toxicology, University of California, Davis, CA USA

**Keywords:** Computational models, DNA-binding proteins

## Abstract

The Ah receptor (AhR) is a ligand-dependent transcription factor belonging to the basic helix-loop-helix Per-Arnt-Sim (bHLH-PAS) superfamily. Binding to and activation of the AhR by a variety of chemicals results in the induction of expression of diverse genes and production of a broad spectrum of biological and toxic effects. The AhR also plays important roles in several physiological responses, which has led it to become a novel target for the development of therapeutic drugs. Differences in the interactions of various ligands within the AhR ligand binding domain (LBD) may contribute to differential modulation of AhR functionality. We combined computational and experimental analyses to investigate the binding modes of a group of chemicals representative of major classes of AhR ligands. On the basis of a novel computational approach for molecular docking to the homology model of the AhR LBD that includes the receptor flexibility, we predicted specific residues within the AhR binding cavity that play a critical role in binding of three distinct groups of chemicals. The prediction was validated by site-directed mutagenesis and evaluation of the relative ligand binding affinities for the mutant AhRs. These results provide an avenue for understanding ligand modulation of the AhR functionality and for rational drug design.

## Introduction

The Ah receptor (AhR) is a ligand-activated transcription factor belonging to the basic helix-loop-helix Per-Arnt-Sim (bHLH-PAS) superfamily of regulatory proteins^1,2^. Binding to and activation of the AhR by a wide variety of exogenous and endogenous chemicals results in the induction or inhibition of diverse gene expression and production of a broad spectrum of toxic and biological effects^3–5^. Additionally, the AhR plays important roles in a variety of physiological responses including developmental and immune processes^6–8^.

A number of studies on the canonical AhR signaling pathway (reviewed in^3,5^) showed that the ligand binds to the PAS-B domain of the AhR which is part of a cytosolic multiprotein complex containing heat shock protein 90 (hsp90), XAP2 and p23. Upon ligand binding, AhR undergoes a conformational change leading to its nuclear translocation, followed by the displacement of AhR-associated proteins by dimerization of the AhR with the homologous ARNT (AhR nuclear translocator) protein and transformation of the AhR into its high-affinity DNA binding form. The binding of the ligand:AhR:ARNT complex to its specific DNA recognition site, the dioxin responsive element (DRE), leads to transcription of the target genes. In addition to this mechanism, several non-canonical pathways related to AhR function have been described^9,10^.

The best characterized ligands for the AhR include halogenated aromatic hydrocarbons (HAHs), such as the polychlorinated dibenzo-p-dioxins, dibenzofurans, and biphenyls, and polycyclic aromatic hydrocarbons (PAHs)^11^. HAHs, such as 2,3,7,8-tetrachlorodibenzo-p-dioxin (TCDD, dioxin) generally have the highest affinities for the AhR and can produce a wide variety of species- and tissue-specific biological and toxic effects. Their toxicity appears to be related to their resistance to metabolism which results in persistent AhR activation^5,12^. In contrast, PAHs and PAH-like chemicals are metabolically labile AhR ligands that produce transient biological effects but no AhR-dependent toxicity^5,12^. In addition to the well known HAH and PAH ligands, numerous natural, endogenous and synthetic AhR agonists and antagonists with diverse molecular characteristics have also been identified, including naturally occurring dietary chemicals (e.g. flavonoids), endogenous indole-containing chemicals (e.g. 6-formylindolo[3,2-b]carbazole (FICZ), indirubin (IR), kynurenines), tetrapyrroles, arachidonic acid metabolites, and other classes of chemicals^11–13^. Studies demonstrating that the AhR also plays a key role in normal physiological functions led to an increased interest in identifying AhR ligands useful as therapeutics (particularly in relation to immunomodulation and cancer)^6,14,15^. A distinct class of AhR ligands (selective AhR modulators (SAhRMs)) have also been identified and they appear to selectively induce a subset of AhR-dependent responses without the major toxic outcomes of dioxins and other HAHs, thus representing intriguing candidates for drug development^6,16,17^.

The identification of AhR ligands with high structural diversity and ability to differently alter the AhR functional activity have promoted detailed studies on ligand binding and activation of the AhR. These studies revealed significant differences in the interactions between structurally diverse ligands and residues within the AhR ligand binding domain (LBD), that account for the observed promiscuity^18^. It has been suggested that these differences may contribute to ligand-specific modulation of the AhR functionality or to alterations of the AhR signaling pathway as a result of ligand-dependent changes in the AhR conformation, or in the structure of either the AhR:ARNT dimer or other AhR:protein complexes^18^. This may lead the activated receptor to interact with different protein partners and/or coactivators, to bind to unconventional DNA sequences, and to differentially alter gene expression^5,10,18^.

An in-depth molecular understanding of differences in ligand binding would provide insights into the key molecular events regulating the mechanisms of AhR activation and transformation and contribute to our understanding of ligand-dependent differences in AhR functionality. Such studies have been hampered for many years by the lack of an experimentally determined three-dimensional structure of the AhR LBD, however, structural models developed by Homology Modeling allowed characterization of the binding cavity and prediction of specific residues that play a critical role in ligand binding [as reviewed in^19^]. Since the experimental structure of the PAS-B domain of the homologous hypoxia-inducible factor 2α (HIF-2α) protein has been available, it has been used as the template for modeling given that it shows the highest degree of sequence identity and similarity with the AhR PAS-B among all the known PAS domains^19^. The first computational studies of ligand binding to the AhR LBD models performed by Molecular Docking allowed prediction of the binding geometries of TCDD and other ligands, interpretation of the observed intra- and inter-species differences in binding, and the virtual screening of potential AhR ligands^19–21^. However, these early computational approaches suffered from several known limitations given by the use of apo template structures for homology modeling and the neglecting of the ligand induced fit effect on the protein conformation^22,23^. Not only did the employment of PAS-B structures of the homologous HIF-2α protein in complex with ligands as templates for modeling greatly improve the quality of the modeled binding site, but the inclusion of a certain degree of receptor flexibility in docking calculations enabled us and other investigators to better describe the binding event^19,24–26^. On the basis of these novel approaches, recent docking studies not only allowed prediction of the binding modes of several AhR ligands but also led to the discovery of novel AhR agonists and SAhRMs^25,27–30^. Moreover they contributed to the study of the role of the AhR in different physiological processes^31,32^.

While several of these studies, coupled with site-directed mutagenesis and functional analysis, have allowed identification of the specific interactions that contribute to the binding of a given ligand within the AhR LBD, a systematic analysis of the binding modes associated with different classes of chemicals is still lacking.

Aim of this work is to identify the differences in the binding modes of ligands with diverse structures and properties within the promiscuous AhR LBD and to unveil the peculiar interactions with residues in the LBD that could have differential effects on the AhR functional mechanism. Therefore, here we present a computational prediction of the binding modes of a group of chemicals representative of the major classes of AhR ligands, and all relatively potent AhR agonists, and demonstrate that they can be classified into different groups according to their specific interactions within the binding cavity. For these purposes we propose a novel computational protocol for molecular docking to the AhR homology model which takes into account receptor flexibility involved in the binding process. Validation of residues predicted to play a critical role in binding of each group of ligands is obtained by site-directed mutagenesis and ligand binding analysis of the mutant AhRs. On the whole, the combination of computational and experimental analyses allowed us to support the hypothesis of differential binding within the AhR binding cavity. This knowledge will allow increased understanding of how each group could yield specific effects on the AhR activation and transformation processes and ultimately contribute to differential modulation of AhR function.

## Results

**Figure 1:**
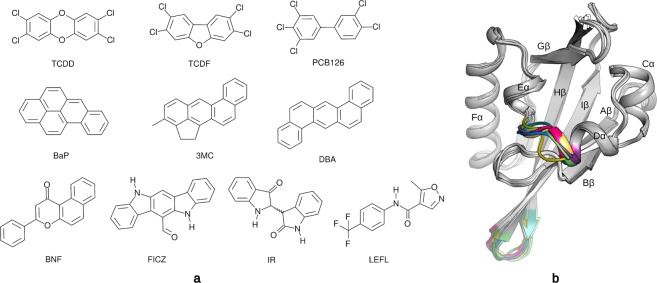
2D representation of the set of ligands analyzed in this study and the homology models of the mouse AhR (mAhR) PAS-B domain developed here. (**a**) Structures of AhR ligands used in this study. (**b**) Cartoon representation of all the mAhR PAS-B models superimposed. The region refined by loop modeling is colored according to the model. Secondary structure elements are labeled according to the PAS domain nomenclature^73^.

To increase our understanding of the molecular determinants of differential ligand binding to the AhR, a set of 10 representative ligands belonging to different chemical classes were selected for detailed analysis (Fig. 1a). TCDD, 2,3,7,8-tetrachlorodibenzofuran (TCDF) and 3,3′4,4′5-pentachlorobiphenyl (PCB126) are HAHs, prototypical and high affinity AhR ligands. While TCDD and TCDF show elongated and planar structures, the shorter PCB126 molecule is characterized by rotation of the chlorinated rings around the central bond, that leads to a higher three-dimensional hindrance. Benzo(a)pyrene (BaP), 3-methylcholanthrene (3MC) and dibenz(a,h)anthracene (DBA) are PAHs characterized by bulky planar structures with extended electron conjugation. BNF is a synthetic flavonoid, FICZ is a photoproduct of tryptophan, IR is a naturally-occurring indole-containing compound, and Leflunomide (LEFL) is an immuno-modulator. In contrast to the very hydrophobic HAHs and PAHs, these latter compounds contain several functional groups that confer a certain degree of polarity to the molecules. All these compounds are known to be AhR agonists^18^.

### All test ligands directly bind to the AhR and stimulate AhR transformation/DNA binding

**Figure 2:**
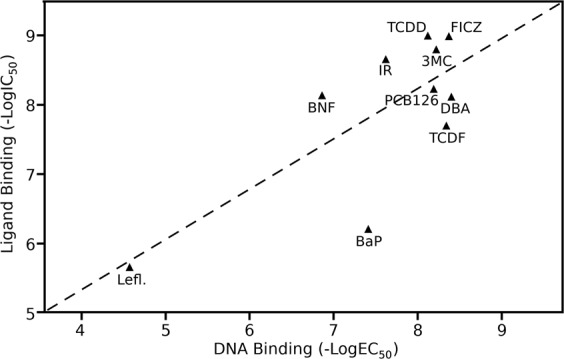
Correlation between the relative affinity of each test compound to bind to the mAhR and their relative potency to stimulate AhR DNA binding (Pearson correlation coefficient R^2^ = 0.56). The relative affinity (LogIC_50_) and potency (LogEC_50_) of the indicated test compounds was determined by [^3^H]TCDD competitive ligand binding and ligand-dependent DNA binding analysis as described under Materials and Methods.

At first, the relative potency of each test compound to bind to the mAhR and stimulate AhR transformation and DNA binding was determined. Ligand binding analysis revealed that all ten ligands could effectively compete with [^3^H]TCDD for binding to the mAhR. The relative affinity of binding of each compound was determined from competitive binding analysis with [^3^H]TCDD and increasing concentrations of each test compound. TCDD and FICZ both demonstrated the highest relative AhR ligand binding affinity (IC_50_) with an IC_50_ of 1 nM, closely followed by 3MC (1.6 nM), IR (2 nM), PCB126 (5.9 nM), BNF (7.2 nM), DBA (7.6 nM), TCDF (20 nM), BaP (617 nM), and LEFL (2190 nM) (Table S1). Similar to ligand binding, all test compounds stimulated transformation/DNA binding of the AhR as determined by gel retardation analysis. Concentration-dependent gel retardation analysis revealed that DBA had the highest relative potency to stimulate AhR DNA binding *in vitro*, with an EC_50_ of 4 nM, followed by FICZ (4.25 nM), TCDF (4.5 nM), 3MC (6 nM), PCB126 (6.5 nM), TCDD (7.5 nM), IR (24 nM), BaP (39 nM), BNF (138 nM), and LEFL (26,800 nM) (Table S1). Overall, as expected, these data not only demonstrated that these chemicals are relatively potent AhR agonists, but that their ligand binding affinity and potency to stimulate AhR transformation/DNA binding were relatively well correlated (Fig. 2).

### Molecular modeling protocol to investigate the molecular determinants of binding

The homology model of the mAhR LBD was developed on the basis of the PAS-B structure of the bHLH-PAS HIF-2α protein, because this domain displays the highest sequence identity and similarity with the target (31% and 52%, respectively) among the available PAS structures. The reliability of the model derived from this template is supported by the observation that the PAS fold is highly conserved, especially among the bHLH-PAS proteins which also share functional similarities^2,33^ (RMSD on Cα lower than 2 Å even between PAS domain pairs with 20–30% identity).

**Figure 3:**
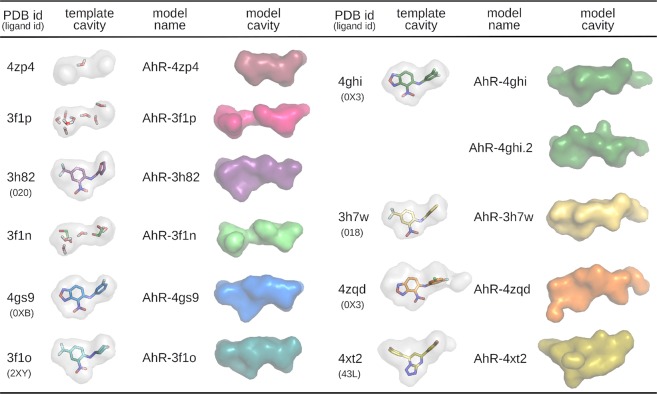
Internal surfaces of the binding cavities for the HIF-2α templates (gray and transparent with the ligand inside) and for the mAhR models (colored and solid). Models are ordered by increasing cavity volumes (Table S3).

To develop homology models of the AhR LBD useful to study binding of ligands with a wide range of diverse structural and physico-chemical characteristics, they must take into account the flexibility and plasticity of the domain, and thus cannot be described with a single structural model. To this end, we built homology models of the mAhR LBD using ten different depositions of the HIF-2α PAS-B, including both apo and ligand-bound structures of the domain (PDB ID: 3F1N:A, 3F1O:A, 3F1P:A^34^; 3H7W:A, 3H82:A^35^; 4GHI:A^36^; 4GS9:A^37^; 4XT2:C^38^; 4ZP4:B, 4ZQD:B^39^). Some of the HIF-2α holo structures were demonstrated to cover configurations relevant for binding AhR ligands. In fact, some ligands co-crystallized with HIF-2α (018 in 3H7W and 020 in 3H82, in Fig. 3, named THS-017 and THS-020, respectively^35^) could competitively displace [^3^H]TCDD specific binding to the mAhR and acted as AhR agonists with affinities and potencies comparable to many well-characterized AhR ligands^24^. In the present study, the most recent HIF-2α holo structures, including the 0XB, 0X3 and 43L ligands (see Fig. 3), were considered as templates to further increase the conformational variability explored in the AhR homology modeling.

During homology modeling, ligands and water molecules present in each HIF-2α PAS-B structure were retained inside the binding cavity to take into account the induced fit effects of the different compounds.

The sequence of the mAhR LBD was aligned to that of the human HIF-2α PAS-B (identical to that of mouse HIF-2α in this region) (Fig. S1) and submitted to MODELLER to develop the comparative models.

For each template, we selected the best among 500 putative models generated with MODELLER using the DOPE score. Refinement by the loop-modeling routine led to 500 loop conformations per model and the best one was again selected using the DOPE score (Table S2). Validation with PROCHECK and ProSa indicated that all were good-quality models (Table S2). For the 4GHI template, two models with comparable DOPE scores were identified and both were included in the analysis.

The structures of the eleven models obtained are very similar in their backbone geometry, including the refined loops (Fig. 1b). What makes each model different from the others are the sidechain conformations that affect the binding cavity volumes (Table S3) and this leads to a wide spectrum of cavity sizes and shapes (Fig. 3). Part of the variability is due to the different ligands present in the template structures and maintained during the modeling step: the smallest cavities derive from the template *apo* structures (4ZP4 and 3F1P) and the largest one from the holo structure with the bulkiest co-crystallized ligand (4XT2).

Ligand preparation (Materials and Methods) included the identification of the most probable tautomeric forms in water solution at pH = 7, performed with Epik. Three possible forms were predicted for IR (trans, cis, and a charged form, Fig. S2) and a unique form for each of the other ligands.

The accuracy of the binding geometries predicted by molecular docking to homology models strongly depends on the quality of the model, particularly in the binding site^22,40,41^. It has been demonstrated that repeating ligand docking to multiple homology models based on different template structures greatly improves docking predictions^40^. More generally, the receptor conformational variability involved in binding can be effectively addressed by docking to an ensemble of static receptor conformations (ensemble-docking technique) derived experimentally, or computationally (*e.g*. by MD simulations), or from protein structure prediction^42^. In this work, to study binding of AhR ligands that have a variety of structural and chemical characteristics, we extended the previously proposed ensemble-docking approach^24^ by including a larger set of different conformations of the AhR LBD (*i.e*. eleven homology models derived from different experimental template structures).

For all the ligands, we obtained several alternative poses depending on the size, shape and conformational characteristics of the receptor model used (Fig. S3). We observed that as the cavity volume increases, the less selective the model becomes. In fact, we obtained docking poses for all the ligands into the largest cavity (AhR-4xt2). In contrast, one model did not produce any docking poses (AhR-3f1p) and three models gave poses only for the smallest ligand, LEFL; these models were derived either from *apo* template structures or have a very small binding cavity (AhR-3h82). Given that we obtained few poses for rigid ligands, regardless of the cavity size, the shape complementarity between ligand and cavity, that was related to the arrangement of internal side-chains, was shown to have a role in determining the ligand binding ability.

Relying on the binding free energy (ΔG_bind_) values, that was calculated with Prime MM-GBSA to obtain an initial rescoring of the docking poses (see Materials and Methods), we selected two poses for each ligand, representative of the variability of the obtained binding geometries. For IR, only the two representative poses obtained for the IR-trans tautomeric form were retained because the trans form is more stable. In some cases (*e.g*. TCDD, TCDF and DBA), the poses of the same ligand differ depending on a translation of the molecule inside the cavity while, in other cases (*e.g*. PCB126, BaP, BNF and LEFL) they differ depending on the ligand rotation of 180° around the minor molecular axis, which inverts the molecular “head” and “tail” (Fig. S4).

Given that neither the docking scores nor the Prime ΔG_bind_ (XP Glide Score and MM-GBSA ΔG_bind(NS)_, in Table S4) were able to clearly discriminate among the putative poses of the same ligand, the two representative poses were further analyzed by Molecular Dynamics (MD) simulations.

While we did not observe the convergence of the MD simulations of the two representative poses into a single pose for most of the ligands, for TCDD, BaP and IR-trans the poses sampled during the two simulations tended to overlap. Moreover, as observed by analyzing the evolution of RMSD from the initial docking pose (Fig. S5), different behaviors occurred during the simulations. Some ligands (TCDF, BNF, LEFL) remain quite close to the docked conformation, while others (BaP, IR, DBA) rapidly change their initial conformation and then remain stable for the rest of the time. Finally, other ligands move away from their initial binding geometry but then move back into their original pose. Despite little changes in ligand positioning can be ascribed to the different force-fields used in the docking and the MD stages, the careful restrained minimization, heating and equilibration stages performed before the MD production runs (as described under Materials and Methods) should have minimized this effect. Therefore, the observed behavior suggested that the modeled AhR conformations used did not completely describe the binding site flexibility (*e.g*. they prevented the AhR ligands from reaching the bottom of the cavity) as a consequence of limitations posed by the template structures. In fact, HIF-2α shows a PAS-B cavity smaller than the AhR one in both the apo and holo structures (Table S3) and its artificial ligands bind mostly in the central part or near to the entrance of the cavity. Thus the final MD refinement allowed to include the local deformations induced by the AhR ligands in the binding cavity.

To compare the two different poses selected for each ligand on an energy base and identify the most stable one, we calculated the ΔG_bind_ with the MM-GBSA method. We used the average value of ΔG_bind_ in the last 8 ns of the simulations (Table S5), which represent a stable portion of simulation for all the complexes (Fig. S5).

Given that all the ligands under study have relatively high affinity for the mAhR and that obtaining a quantitative prediction of the binding free-energy is beyond the aim of this work, we did not expect to find a good correlation between the computed MM-GBSA ΔG_bind_ and the experimental binding affinities. The lack of correlation obtained using the best docking score (R^2^ = 0.03) was improved using the Amber MM-GBSA ΔG_bind_ values (Pearson R^2^ = 0.37 and Spearman ρ = 0.64), which indicated a weak trend (Fig. S6).

The pose with the most favorable ΔG_bind_ was further analyzed to rationalize the binding mode.

### Characterization of binding modes and interactions of the different ligands

**Figure 4:**
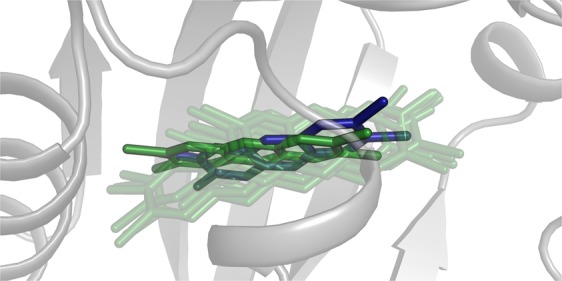
Dynamic view of a TCDD pose inside the binding cavity; ligand is shown as sticks and protein as gray cartoons. In blue, the pose previously obtained by docking^24^; in dark green, one of the docking poses obtained in this work; in green, 10 snapshots taken from the last 8 ns of the MD simulation.

MD simulations allowed us to obtain an improved description of the ligand binding modes and the identification of all the interacting residues^21,24,43^. The new dynamic representation of TCDD binding here obtained can be compared to the static view obtained by previous docking calculations and validated by extensive mutagenesis studies^21,24^ (Fig. 4). During MD, TCDD moves inside the cavity by translating from the inner part to the center of the cavity and samples also the previously determined pose, lying in an average position compared to those obtained by MD.

**Figure 5:**
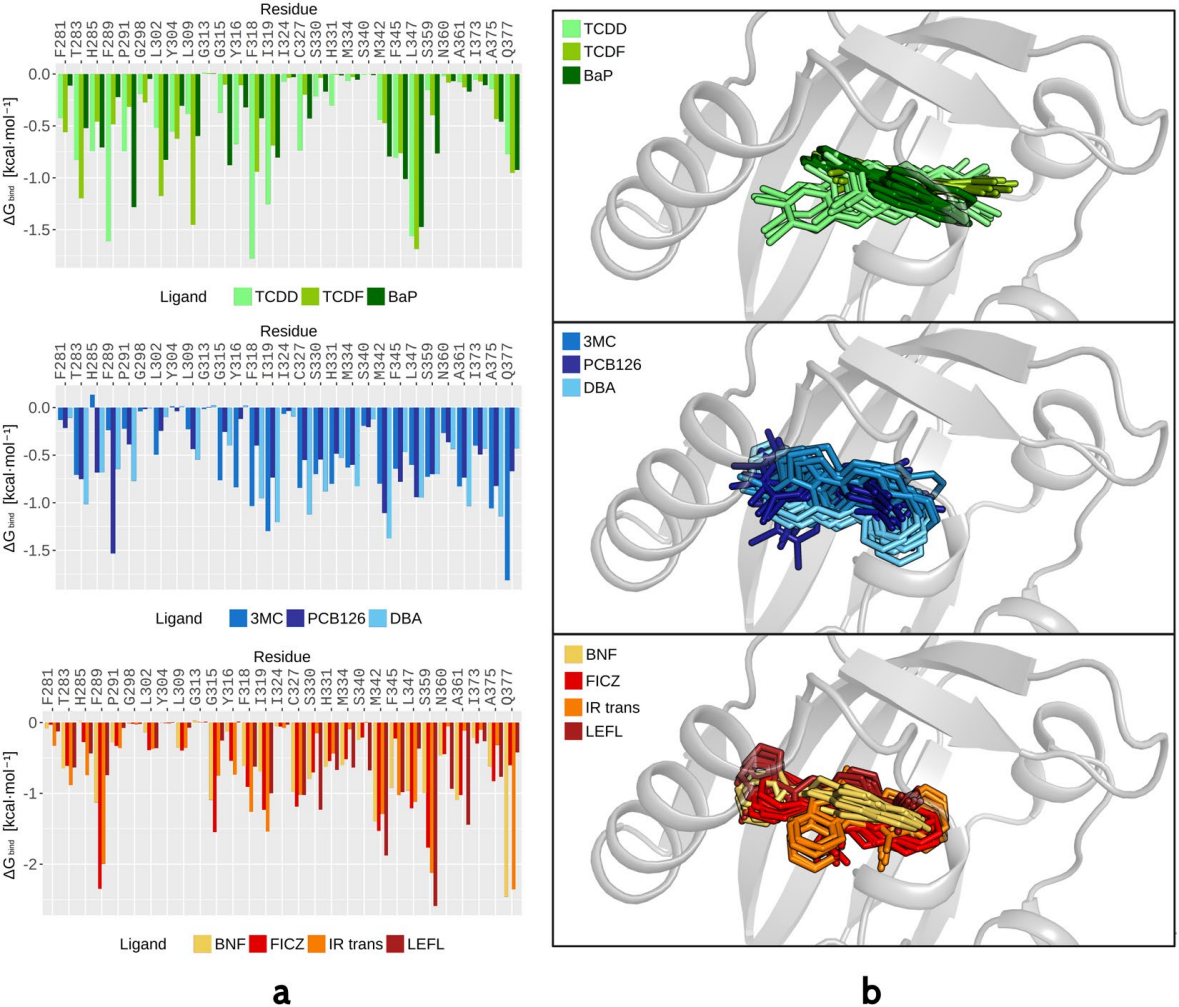
Ligands are gathered into three groups that were defined by the occupancy of different sites inside the AhR cavity and by characteristic residue interactions during MD simulations. (**a**) Per-residue decomposition of ΔG_bind_. Values were obtained as averages of the last 8 ns of simulation. In the plots, only residues lining the internal cavity are shown. (**b**) Ten snapshots sampled during the last 8 ns of simulation are shown for each ligand belonging to the three groups; ligands are represented as sticks.

By analyzing the different profiles obtained by per-residue decomposition of the ΔG_bind_ for the best pose (the one with the lowest ΔG_bind_, Table S5) for each of the ligands under study (Fig. 5a) it was possible to identify a set of residues that stabilize all the ligands, with most of them belonging to the previously published “TCDD-binding fingerprint”^21^. In the present study we especially highlighted the importance of T283, F289, F318, I319, F345, L347 and Q377. Additional interactions that characterize binding of each ligand were identified and used to gather ligands into three different groups (Fig. 5).

The first group includes TCDD, TCDF and BaP, and these ligands bind at the bottom of the cavity (Bβ, Cα, Dα region) and are stabilized by hydrophobic interactions. In the second group, 3MC, PCB126, and DBA bind nearer to the Fα/Gβ site and also have hydrophobic interactions. Ligands of the third group (BNF, FICZ, IR, LEFL) lie in the same position as group 2, but establish hydrogen-bonds and polar interactions with several residues (Fig. 5).

**Figure 6:**
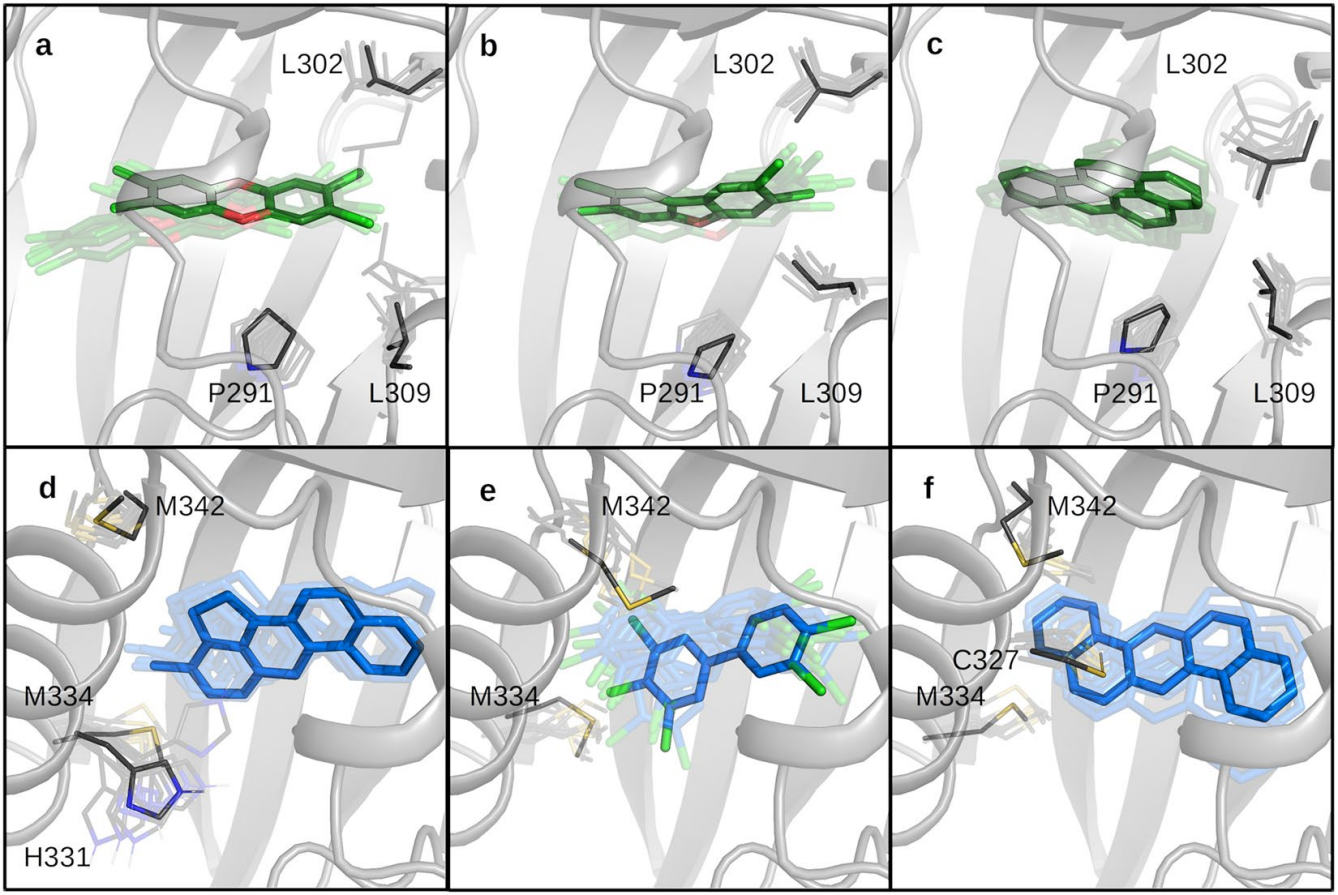
Dynamic view of the binding poses of ligands in group 1, (**a**) TCDD, (**b**) TCDF, (**c**) BaP and group 2, (**d**) 3MC, (**e**) PCB126, (**f**) DBA. Ligands are represented as sticks and 10 snapshots extracted during the last 8 ns of MD simulation are shown in transparency; solid sticks indicate the most sampled poses in the MD simulations. The most relevant residues identified by per-residue decomposition of ΔG_bind_ are shown as lines and labeled.

Beside the hydrophobic interactions common to all the ligands of this study, in the central part of the cavity (F289, F318, I319, F345 and L347), ligands in group 1 are stabilized by additional interactions with residues at the bottom of the cavity (L302, L309 and P291 (Fig. 6)). As already pointed out, TCDD moves inside the cavity (Fig. 6a) and reaches these residues. Similar movements are observed for TCDF (Fig. 6b), but it mostly occupies the Cα/Dα site as in the original docking pose, as well as for BaP (Fig. 6c). Ligands in group 2 are very stable in the Fα/Gβ site (Fig. 6), except for PCB126 that also moves to the center of the cavity, where it assumes planar conformations (Fig. 6e). The two residues that characterize these binding modes are M334 (on Fα) and M342 (on Gβ). As evident in the per-residue ΔG_bind_ profiles (Fig. 5a), group 2 stabilization can be attributed to many residues in the whole cavity (except in the zone at the bottom of the cavity). In fact, 3MC and DBA are bulky ligands and tend to occupy almost the entire volume of the cavity, and PCB126 can contact many residues due to its conformational flexibility around the central bond and the hindrance of five chlorine atoms. In some simulations, the H331 histidine sidechain, that flips inside the cavity, gives stabilizing interactions with the ligand (e.g. with 3MC (Fig. 6d)).

**Figure 7:**
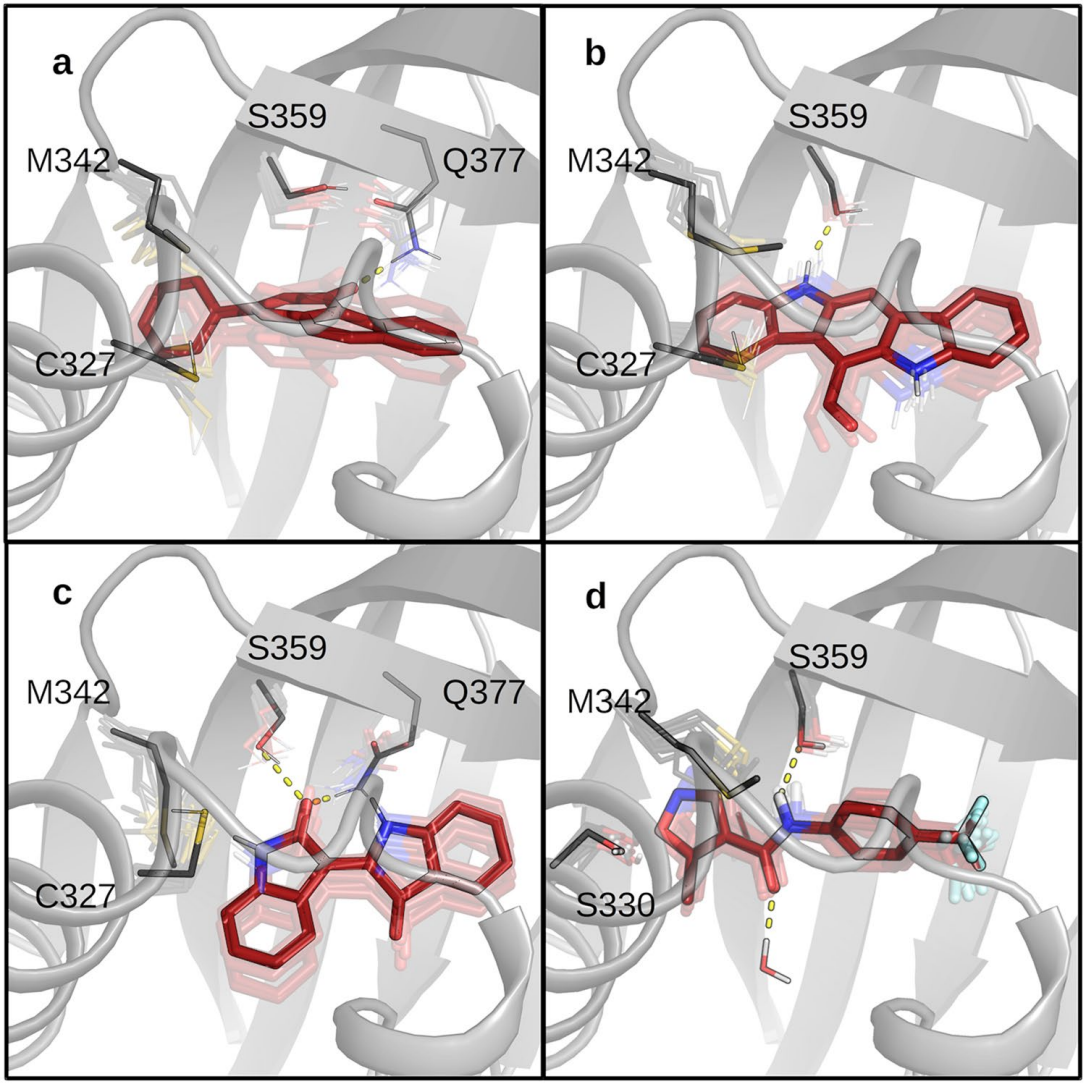
Dynamic view of the binding poses of ligands in group 3: (**a**) BNF, (**b**) FICZ, (**c**) IR, and (**d**) LEFL. Ligands are represented as sticks and 10 snapshots extracted during the last 8 ns of the MD simulation are shown in transparency; solid sticks are the most sampled poses in the MD simulations. The most relevant residues identified by per-residue decomposition of ΔG_bind_ are shown as lines and labeled.

Group 3 is the most heterogeneous, and like group 2, ligands occupy the Fα/Gβ site (Fig. 7) where they are stabilized by interactions with some residues belonging to Fα (C327 or S330) and Gβ (M342). The peculiarity of this group is given by the polar interactions and hydrogen-bonds established by ligands with two internal sidechains, Q377 and S359. Given that the mAhR cavity is hydrophobic, polar residues (S359, Q377, T283, H285) tend to interact with each other or with water molecules by hydrogen bonding.

While some ligands are able to interfere with this interaction chain (BNF and IR in Fig. 7a,c), others act as hydrogen-bond donor toward S359 while preserving the H-bond between S359 and Q377 (FICZ and LEFL, Fig. 7b,d). For LEFL we also observed a water-bridged H-bond between the oxygen atom of its amidic group and the hydroxyl of Y316.

To obtain an experimental validation of the different binding modes predicted by our analysis, for each group of ligands we selected a small set of residues to be tested in mutagenesis studies. Non-selective residues that give stabilizing interactions with all the ligands under study were not included in this validation. Therefore, for group 1 we selected L302, L309 and P291, for group 2, M334 and M342 (in addition H331 for 3MC, and C327 for DBA), and for group 3, C327, M342 and S359 (in addition S330 for LEFL).

### Targeted point mutations within the AhR alter ligand binding affinities

To examine the role of each of these targeted amino acids in ligand-selective AhR activation, we initially determined the effect of their mutation to alanine (L302A, L309A, C327A, H331A, M334A, M342A, and S359A) or leucine (P291L) on ligand-dependent transformation/DNA binding of *in vitro* synthesized AhR. These analyses demonstrated that six of the eight amino acid mutations (P291L, C327A, H331A, M334A, M342A, and S359A) resulted in ligand-dependent AhR:ARNT:DRE specific complex formation greater than 50% of wt/mAhR activated by TCDD; the L302A mutation eliminated ligand-stimulated AhR DNA binding and the L309A substitution resulted in less than 25% TCDD-induced AhR DNA binding and little to no TCDF- or BaP-induced AhR DNA binding (Fig. S7). Therefore L302A and L309A could not be used for the subsequent competitive binding analysis.

**Figure 8:**
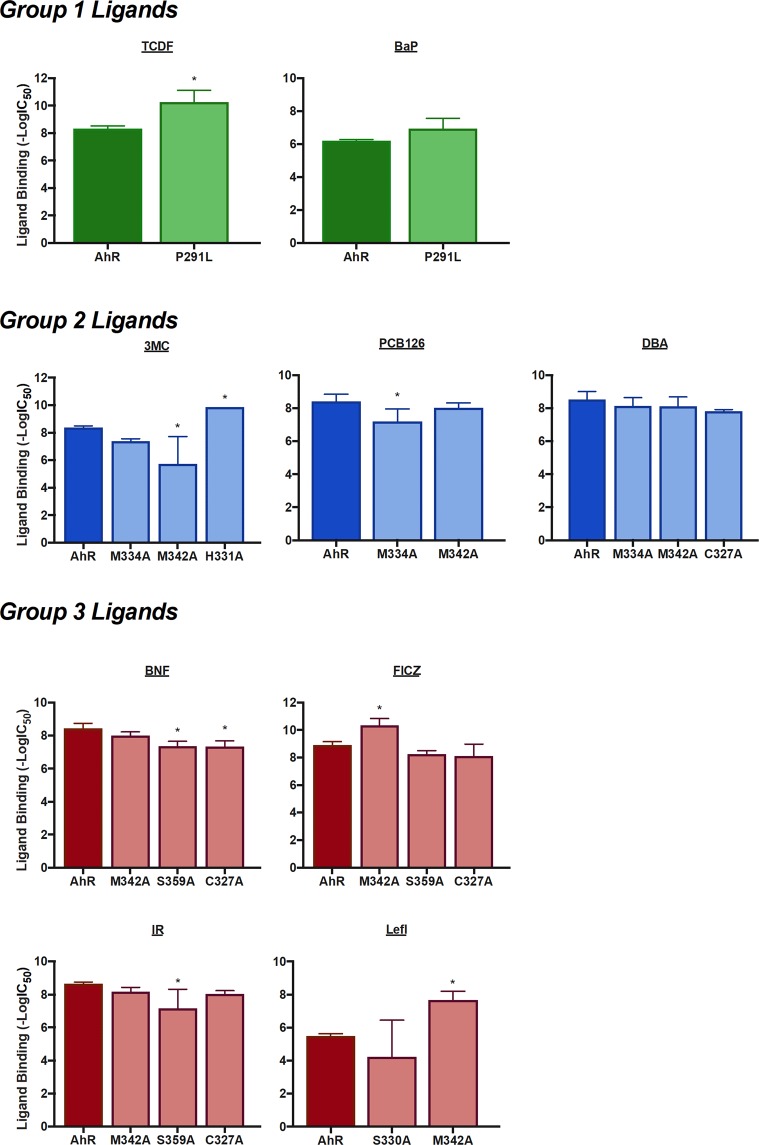
Relative binding affinity for group 1, 2 and 3 ligands relative to wild-type and mutant AhRs. The relative affinity (−logIC_50_) of each test chemical for the AhR ligand was determined from concentration-dependent inhibition curves obtained using [^3^H]TCDD ligand binding analysis, as described under Materials and Methods. The mean IC_50_ ± standard deviation was determined using three-parameter non-linear regression of nine independent reactions. (*) Represents a significant (p ≤ 0.05) change in ligand binding affinity relative to wild-type mAhR in One-Way ANOVA Multiple Comparison Test.

To assess the influence of the remaining six residues in binding diverse ligands within the AhR ligand binding pocket, [^3^H]TCDD competitive ligand binding was carried out with increasing concentrations of each ligand and their relative affinity (IC_50_) calculated from the competitive binding curves (Table S6 and Fig. 8). Interestingly, ligand binding analysis revealed that P291L substitution dramatically enhanced the relative affinity of TCDF for the AhR (compare 20 nM for wild-type (wt) AhR (Table S1) to 0.04 nM for the P291L AhR (Table S6)), but while the mutation suggests an increase in BaP binding, the result was not statistically significant. The relative binding affinity of PCB126, IR and 3MC were reduced with the M342A substitution, but the relative affinity of LEFL and FICZ were significantly increased. In contrast, DBA and BNF were not affected by the M342A mutation. The M334A substitution significantly reduced the relative affinity of PCB126 and decreased 3MC binding, but had no significant effect on the binding of DBA. Interestingly, while the H331A mutation dramatically increased the relative affinity of 3MC for the AhR, S330A had no significant effect on LEFL AhR binding. The results with the S359A substituted AhR were similar to that of M342A in that it significantly increased the relative binding affinity by one ligand (BNF) and a decreased binding of another (FICZ). Similarly, the C327A mutation significantly decreased BNF, but had no significant effect on that of IR, DBA or FICZ. Overall, the results of these mutational analyses reveal significant differences in ligand-specific, amino acid-dependent binding to the AhR.

## Discussion

Computational and experimental analyses of AhR binding to a set of known AhR ligands with different structural and physico-chemical properties allowed us to classify these compounds into three groups according to their binding characteristics. An in-depth analysis of the molecular determinants of binding of each compound required the development of an adequate computational protocol able to provide a dynamic view of the process as well as an extensive site-directed mutagenesis plan coupled with evaluation of the binding affinity of each mutant for the selected chemicals to validate the predictions.

For TCDD, TCDF and BaP, characteristic contributions to the ΔG_bind_ are provided by interactions with L302 and L309 at the bottom of the cavity (Fig. 6a–c). While previous docking analyses indicated the TCDD placement at the center of the cavity^20,21,24–26,44^, our MD refinement of the docking poses suggested that TCDD can translate from the center to the inner part of the cavity and vice versa (Fig. 4) thanks to a local deformation of the binding cavity induced by this ligand. Also the initial docking pose of BaP, that we found in the center of the cavity consistently with other docking studies^44^, was dramatically changed by MD simulations, that allowed the ligand to reach the bottom of the cavity. Conversely, TCDF was already predicted to reach the inner region by docking calculations, and MD refinement did not alter this placement.

We previously observed that the L302A, L309A mutations dramatically reduced TCDD binding activity, whereas C327A, M334A and S359A, at the entrance of the cavity, only partially affected it^24^. In the present work we demonstrated that these two mutations eliminated or greatly reduced the AhR DNA binding induced by TCDD, TCDF and BaP (Fig. S7). Given the good correlation observed between binding affinity and potency to stimulate AhR DNA binding (Fig. 2) we could confirm the role of the long hydrophobic leucine sidechains in the inner part of the cavity in stabilization of these three ligands. Conversely, the IC_50_ data (Fig. 8) for the P291L mutant revealed that indeed P291, lying at a lateral side of the hydrophobic region, is not deeply involved in the stabilization of this group of ligands. In fact, this mutation had limited effect on BaP binding and the observed enhancement of the TCDF affinity could be related to the more effective stabilization produced by the longer leucine sidechain.

The second group of chemicals includes 3MC, PCB126 and DBA. Our computational protocol predicted that the two bulky PAHs tend to occupy almost the entire space within the cavity, except the inner region, with a number of stabilizing hydrophobic interaction with the internal sidechains (Fig. 5) and the PCB126 occupies the same region thanks to its conformational flexibility. All the three ligands showed characteristic interactions with hydrophobic residues, M334 and M342, at the Fα/Gβ site. Moreover, 3MC contacts the flexible sidechain of H331, on the Fα, several times during the MD simulation (Fig. 6d–f).

The lower binding affinities of 3MC and PCB126 for the M334A and M342A mutants in comparison with the wt AhR (Fig. 8) confirmed the hypothesis that substitution of the long hydrophobic sidechains with alanine decreases stabilization of the two ligands. Contrary to expectations, the H331A mutation increased the 3MC affinity. This is probably due to the removal of unfavorable interactions with a polar sidechain. Evaluations of IC_50_ for DBA binding to M334A and M342A (Fig. 8) revealed that computational predictions in that case were incorrect; in fact, the DBA binding affinity for the wt AhR remained unaltered upon both mutations. It is conceivable that the putative docking pose for DBA, in which molecule reaches the inner part of the cavity (similarly to group 1 ligands), that was discarded during the selection of the best pose, might be more reliable.

The binding poses of the third group of ligands, including BNF, FICZ, IR and LEFL, were predicted at the Fα/Gβ site similarly to ligands of group 2, but their stabilization derived from different contributions, *i.e*. electrostatic interactions and hydrogen-bonds with the polar residues in this region: C327, S330, S359, Q377 (Fig. 7). MD refinement indicated that these strong interactions determine poor mobility of the ligands into the cavity and revealed that while BNF and IR are able to break an inter-residue H-bond between S359 and Q377, FICZ and LEFL only partially interfere with the same H-bond network.

Given that the mutation of Q377 to alanine was proved to abolish TCDD specific binding^43^, this mutant could not be used to evaluate the relative affinity of BNF and IR by competitive binding analysis with [^3^H]TCDD. However, the observed reduction of the affinities of BNF and IR for the S359A and C327A mutants compared to the wt AhR (Fig. 8) confirmed both the predicted binding poses and the key role of the electrostatic interactions in ligand stabilization. While the S359 residue appeared to play a less important role in stabilization of FICZ binding, the observed higher affinity of this ligand for M342A compared to the wt AhR (Fig. 8) could confirm its placement in the same binding site of the other ligands of this group. In fact, it is conceivable that the long hydrophobic methionine sidechain in the Gβ strand could interfere with ligand stabilization. Noticeably, experiments to determine the relative binding affinity of LEFL for S359A from concentration-dependent inhibition curves resulted in nonconvergence (Table S6), thus indicating a very low affinity of LEFL for this mutant in agreement with our prediction. A further confirmation of the LEFL binding pose could derive from the significant increase of LEFL binding affinity upon mutation of M342 to alanine, similar to that observed for FICZ (Fig. 8).

While other investigators predicted binding characteristics similar to those here described for FICZ and IR using different docking approaches, they suggested that these ligands share the same binding site of TCDD^20,25,26^. Here we demonstrated that while TCDD can reach the inner hydrophobic region of the AhR cavity thanks to induced-fit effects, FICZ, IR and the other group 3 chemicals bind nearer to the entrance of the cavity and form H-bonds with residues in this region.

Another confirmation of differential binding of TCDD (group 1) and LEFL (group 3) ligands derives from a study on ligand binding to different zebrafish AhR paralogues (isoforms: 1a, 1b and 2), where it was found that the zebrafish AhR1a (zfAhR1a) was unable to bind TCDD but could still bind and be activated by LEFL^45^. Our previous comparative analysis of the AhR LBDs homology models of different species revealed that zfAhR1a has a dramatically shortened binding cavity compared to the mouse AhR^46^ and this is due to the sidechains of three residues at the center of the cavity, which reduce the available internal space to only the region at the entrance of the cavity (Fα/Gβ site). A confirmation of the role of this reduction on differential binding was provided by the evidence that mutation of these residues in zfAhR1a to those present in mouse AhR restored the zfAhR1a ability to bind TCDD^46^. These findings, in addition to the mutagenesis data here presented, strongly confirm our prediction that the binding site of LEFL (and of the other group 3 chemicals) is located near to the entrance of the AhR binding cavity.

## Conclusions

Our novel approach for ligand docking to the AhR homology models was very effective in capturing the differences in binding of diverse agonists. Structure-driven site-directed mutagenesis followed by evaluation of the relative ligand binding affinities for the obtained mutants provided confirmation of most of the computational predictions.

Among the three groups with different binding sites and interactions, the main difference was observed between groups 1 and 3, that show completely different physico-chemical characteristics and accordingly yield interactions with hydrophobic residues in the inner part of the cavity (group 1, including TCDD, TCDF and BaP) or with polar residues at the Fα/Gβ site (group 3, including BNF, FICZ, IR and LEFL). Another characteristic predicted for group 3 chemicals was the ability to break an inter-residue H-bond network existing at the center of the cavity. An additional determinant of group 1 binding was their ability to penetrate deeply into the cavity using the flexibility and plasticity of the inner zone of the cavity. In contrast, 3MC and PCB126 (group 2), despite the hydrophobic stabilization similar to that of group 1, cannot reach the inner part of the cavity due to their high steric hindrance and thus they occupy a binding site nearer to the entrance of the cavity.

While we were able to demonstrate differences in ligand binding of this set of chemicals, what remains to be determined is whether the different pattern of interactions with residues within the AhR binding cavity can result in differential effects on AhR conformational changes and interactions with protein partners that may propagate downstream in the AhR signaling pathway. It is conceivable that ligands that bind near to the entrance of the cavity could have a more favorable dissociation kinetics (high k_off_ constants) that could lead to a lower stability of the complexes or reduced rates of AhR transformation. On the other hand, our modeling studies on the AhR:ARNT dimer, based on the HIF-2α:ARNT template, predicted that the Fα/Gβ site may be involved in the dimerization interface^47,48^. Therefore, specific perturbation effects on the dimer structure and stability could be produced by ligands that are in contact with this interface, similarly to what suggested for the HIF-2α:ARNT dimer^49^. These and other hypotheses will be addressed in future studies aimed at analyzing the effects of differential ligand binding on the ligand-specific AhR transformation and dimerization. Understanding the effects of differential binding in modulating the AhR functionality would allow the design of new ligands targeted to promote specific alterations of the AhR mechanism useful for medicinal chemistry and therapeutic applications.

## Materials and Methods

### Chemicals

TCDD was obtained from Dr. Stephen Safe (Texas A&M University), [^3^H]TCDD (14.3 Ci/mmol) was obtained from ChemSyn Laboratories (Lenexa, Kansas), and TCDF, PCB126 and DBA were from Accustandard (New Haven, CT). [^32^P]-ATP (~6,000 Ci/mmol) was from Perkin Elmer Life & Analytical Sciences. The structures of the specific AhR ligands used in these studies are shown in Fig. 1a. 3MC, BNF, LEFL, and dimethyl sulfoxide (DMSO) were from Sigma-Aldrich (St. Louis, MO) and FICZ were from Tocris Bioscience (Minneapolis, MN) and IR from AmplaChem (Carmel, IN). All chemical stocks and dilutions were prepared in DMSO.

### Plasmids

The mouse AhR (mAhR) and ARNT (mARNT) expression plasmids, mβAhR/pcDNA3 and mβArnt/pcDNA3, have been previously described^50^. Point mutations of mβAhR/pcDNA3 were carried out using the QuikChange Lightning Mutagenesis Kit (Agilent Technologies) and all constructs were verified by sequencing.

### Homology modeling

The structures of the selected chains of the templates were downloaded from the Protein Data Bank (PDB) and prepared using the Protein Preparation Wizard included in the Schrödinger suite. This tool adds the hydrogen atoms, optimizes the hydrogen bond network and minimizes the energy with the OPLS3 force field. All the atoms inside the cavities were kept (ligands for *holo* structures and water molecules for *apo* structures).

The target-to-template sequence alignment was obtained using MUSCLE^51^. The homology models were developed using MODELLER^52^, that implements an approach to comparative modeling by satisfying spatial restraints. During modeling, ligands and water molecules included in the template structures were copied into the target models using the BLK function of MODELLER. The loop-model routine implemented in MODELLER, that combines optimization steps and molecular dynamics with simulated annealing^53^, was used to refine the loops. The Discrete Optimized Protein Energy (DOPE) statistical potential^54^ was used to select the best model. The obtained models were then prepared with the Protein Preparation Wizard, after removal of ligand and water molecules, and were subjected to energy minimization with MacroModel. In this last step, the sidechains of residues lining the cavity, identified by CASTp^55^, were free to move, while their backbone and the rest of the protein were constrained by a force constant of 200 kJ·mol^−1^·Å^−2^. The quality of the final models was assessed using PROCHECK^56^, that provides information about the stereochemical quality, and by the ProSA validation method^57^ that evaluates model accuracy and statistical significance with a knowledge-based potential. The G-factor from PROCHECK provides a measure of how unusual the overall stereochemical properties are. Ideally it should be near to 0, structures associated to values below −0.5 are considered unusual and below −1.0 highly unusual^58^. The Z-score from ProSa indicates overall model quality and the reference ranges change depending on the size of the protein; in case of a 108 amino acid protein the range of normality is approximately between −7.5 and 0^57^.

### Molecular docking

The structures of the ligands were downloaded from PubChem and then prepared with the LigPrep utility in the Schrödinger suite. Their protonation states were determined with the Epik tool for pKa prediction included in Maestro^59^, that is based on PROPKA as heuristic pKa calculator. Ligand structures were then optimized using MacroModel with the OPLS3 force field in implicit water.

Docking was performed using Glide XP (extra precision)^60^ included in the Schrödinger suite 2016–3. This method uses a hierarchical series of filters to search for possible locations of the ligand in the binding site and includes a flexible treatment of the ligand. The shape and properties of the protein are represented on a grid by different sets of fields that provide progressively more accurate scoring of the ligand poses. Glide XP performs extensive sampling for ligand positioning through an anchor-and-grow approach and also accounts for explicit waters. The method uses a scoring function (XP GlideScore) that includes force-field-based functions to describe Coulomb and van der Waals contributions to the interaction energy as well as empirically-based functions. In this work, grids for mAhR homology models were set up using default parameters and the binding box was centered in the center of mass of three internal residues (H285, S359, Q377) with a 10 Å side length.

### Molecular dynamics simulations

Each selected docking pose was prepared for simulation using the tleap module of the AMBER14 package^61^ and the ff14SB^62^ force field with TIP3P water placed up to 12 Å from the solute and neutralizing the system with 7 Cl− ions. Parametrization of the ligands was performed using the Antechamber module of AMBER14, using the Generalized Amber Force Field^63^ (GAFF) to assign the atom-types and the AM1-BCC method^64^ to assign charges. A prior multistage equilibration approach was used to remove unfavorable contacts and provide a reliable starting point for the simulations. The systems were subjected to 1000 steps of steepest descent energy minimization, followed by 1000 steps of conjugate gradient with restraint applied to backbone and ligand atoms (100 kcal mol^−1^ Å^−1^). Subsequently, a 750 ps MD simulation was used to gradually heat the system from 0 to 100 K in the NVT ensemble with backbone restraint lowered to 10 kcal mol^−1^ Å^−1^ and from 100 to 300 K in NPT ensemble with backbone restraint lowered to 2 kcal mol^−1^ Å^−1^. Finally, the systems were equilibrated with a 1.0 ns NPT simulation mantaining the backbone restraint of 2 kcal mol^−1^ Å^−1^. All the restraints were removed for the production runs. In all the stages, the temperature was controlled by the Langevin temperature equilibration scheme with a collision frequency of 2.0 ps^−1^ and pressure targeted to 1 bar using a Berendsen barostat. A time step of 2.0 fs was used, together with the SHAKE algorithm to constrain the bonds connecting the hydrogen atoms. The Particle Mesh Ewald method was used to treat the long-range electrostatic interactions with the cutoff distances set to 9 Å. Production runs were carried out for 10 ns in the case of some ligands, and 20 ns in the case of other ligands, to allow a complete equilibration of the different poses.

MD trajectories were visually inspected using VMD^65^. Images were generated with Pymol.

### Binding free energy calculations

The binding free energy (ΔG_bind_) for complex formation was evaluated by means of the Molecular Mechanics Generalized Born Surface Area (MM-GBSA) method that adopts an implicit solvent model^66^.

In particular, the Prime MM-GBSA method^67^ was used for an initial rescoring of the docking poses. In this case, the ΔG_bind_ is obtained as the sum of energy associated with complex formation in the gas-phase, calculated with OPLS3 force field, and the difference in solvation free energies between the complex and the unbound monomers, calculated using the VSGB solvation model^68^. We used the Single Frame Protocol in Prime (shorten NS), *i.e*. both ligand and protein conformations were obtained from the optimized structure of the complex instead of performing distinct optimizations of the three different states (ligand, protein, and complex).

Moreover, the MM-GBSA method implemented in the AMBER software package was applied to the MD trajectories of the two representative poses of each ligand to evaluate their relative stability. The polar solvation term was approximated with the Generalized Born (GB) model using OBC re-scaling of the effective Born radii^69^. The non-polar solvation term was calculated as the product of the surface tension parameter and the solvent accessible surface area (SA) evaluated using the Linear Combination of Pairwise Overlap (LCPO) algorithm^70^. The single-trajectory approach was selected, *i.e*. the conformational ensemble was extracted from the single trajectory of the complex, instead of the three-trajectory approach (that uses the separate trajectories of the complex, receptor and ligand). The per-residue energy decomposition analysis was used to extract the contributions of single residues to the ΔG_bind_, and those of residues within the binding site allowed identification of the hotspot residues for the different ligands. An ensemble of 500 conformations regularly sampled in the last 8 ns of the different simulations was used both in calculations of the ΔG_bind_ of the simulated poses and in the per-residue decomposition.

### Hydroxyapatite [^3^H]TCDD ligand binding assay

[^3^H]TCDD specific binding to wild-type or mutant mAhRs synthesized *in vitro* using the Promega TNT Quick coupled transcription/translation rabbit reticulocyte lysate kit (Madison, WI) was carried out as previously described^50^. [^3^H]TCDD specific binding was determined by subtracting the amount of [^3^H]TCDD bound to unprogrammed lysate (nonspecific binding) from the total amount of [^3^H]TCDD binding to lysate containing *in vitro* expressed AhR. The amount of [^3^H]TCDD specific binding remaining in the presence of the indicated competitor chemical was expressed as a percent of the total [^3^H]TCDD specific binding. The relative binding affinity (IC_50_) of each chemical was determined from concentration-dependent competitive inhibition curves obtained using [^3^H]TCDD and increasing concentrations of each test chemical and the mean IC_50_ value was determined using three-parameter non-linear regression.

### AhR DNA binding (Gel Retardation) assay

Wild-type and mutant mAhRs and mARNT were synthesized *in vitro* in the presence of unlabeled L-methionine, the resulting mAhRs and mARNT translation mixtures and MEDGK (25 mM MOPS (3-(N-morpholino)propanesulfonic acid; pH 7.5), 1 mM EDTA, 1 mM dithiothreitol, 10% (v/v) glycerol, 150 mM KCl) were mixed in a 1:1:8 (v/v/v) ratio and incubated with DMSO (1% final concentration) or the indicated concentration of TCDD or test chemical for 2 hours at room temperature. An aliquot of each incubation was mixed with [^32^P]-labeled double-stranded oligonucleotide containing the mouse AhR DNA binding site DRE3 (a dioxin responsive element from the upstream region of the murine Cyp1a1 gene^71^) and protein-DNA complexes resolved by gel retardation analysis as previously described in detail^72^. Gels were visualized using a FLA9000 Fujifilm Imager and protein-DNA complexes quantitated with Fujifilm MultiGauge software. For potency measurements, reactions were incubated with increasing concentrations of the test chemical and the chemical concentration producing half maximal AhR:ARNT:DRE complex formation (EC_50_) determined.

## Supplementary information

SGianiTagliabue-SupplInfo
