# Supplementary material for: Modeling the binding of diverse ligands within the Ah receptor ligand binding domain
Source: Sci Rep. 2019 Jul 23;9:10693. doi: 10.1038/s41598-019-47138-z (PMC6650409; doi:10.1038/s41598-019-47138-z)
Supplement: Supplementary file 1 — SGianiTagliabue-SupplInfo [file 41598_2019_47138_MOESM1_ESM.pdf]

Modeling the binding of diverse ligands within the Ah receptor ligand binding domain

Sara Giani Tagliabue, Samantha C. Faber, Stefano Motta, Michael S. Denison, and Laura Bonati*

Supplementary information

Table S1. Relative affinity and potency of test chemicals determined in AhR ligand binding (AhR LB) and DNA binding (AhR DNAB) assays. The relative potency of each test chemical for AhR ligand binding (IC_{50}) and transformation/DNA binding (EC_{50}) was determined from concentration-inhibition or concentration-response curves obtained using [^{3}H]TCDD ligand binding and gel retardation analysis, respectively, as described under Materials and Methods. The mean IC_{50}/EC_{50} ± standard deviation was determined using three-parameter non-linear regression with nine independent reactions.

Ligand id	AhR LB IC_{50} [M]	AhR LB SD [M]	AhR LB -LogIC_{50}	AhR DNAB EC_{50} [M]	AhR DNAB SD [M]	AhR DNAB -LogEC_{50}
TCDD	1.00E-09	-	9.00	7.53E-09	1.10E-09	8.12
TCDF	2.01E-08	2.24E-09	7.70	4.55E-09	5.37E-10	8.34
PCB126	5.89E-09	3.29E-09	8.23	6.51E-09	9.45E-10	8.19
BaP	6.17E-07	8.41E-08	6.21	3.89E-08	4.65E-09	7.41
3MC	1.58E-09	1.27E-09	8.80	6.00E-09	4.87E-10	8.22
DBA	7.60E-09	4.20E-09	8.12	4.02E-09	3.80E-10	8.40
BNF	7.24E-09	2.30E-09	8.14	1.38E-07	3.06E-08	6.86
FICZ	1.02E-09	7.86E-10	8.99	4.25E-09	5.85E-10	8.37
IR	2.17E-09	1.02E-09	8.66	2.39E-08	9.90E-09	7.62
LEFL	2.19E-06	1.03E-06	5.66	2.68E-05	1.75E-06	4.57

Table S2. DOPE score and validation scores of PROCHECK and ProSA for the mAhR models

model	DOPE score	PROCHECK G-factor*	ProSA Z-Score**
AhR-4zp4	-1146.24	-0.15	-3.57
AhR-3f1p	-1222.75	-0.14	-4.11
AhR-3h82	-1216.72	-0.30	-4.10
AhR-3f1n	-1213.07	-0.19	-4.03
AhR-4gs9	-1192.83	-0.25	-4.23
AhR-3f1o	-1186.26	-0.26	-4.11
AhR-4ghi	-1160.52	-0.35	-4.13
AhR-4ghi.2	-1159.28	-0.35	-3.98
AhR-3h7w	-1283.28	-0.22	-4.27
AhR-4zqd	-1153.25	-0.24	-3.57
AhR-4xt2	-1187.60	-0.36	-3.59

* PROCHECK G-factor values are "normal" if ≥ -0.5

** ProSA normal Z-score ranges between -7.5 and 0

Table S3. Comparison of the internal cavity volumes (calculated by CASTp) in the HIF-2α template structures and in the mAhR models

PDB id	template volume (Å^3)	ligand id	model name	model volume (Å^3)
4zp4	375	water	AhR-4zp4	417
3f1p	364	water	AhR-3f1p	478
3h82	439	020	AhR-3h82	489
3f1n	383	EDO	AhR-3f1n	491
4gs9	424	0XB	AhR-4gs9	533
3f1o	425	2XY	AhR-3f1o	568
4ghi	443	0X3	AhR-4ghi	575
4ghi	443	0X3	AhR-4ghi.2	629
3h7w	421	018	AhR-3h7w	689
4zqd	569	0X3	AhR-4zqd	712
4xt2	561	43L	AhR-4xt2	808

Table S4. XP Glide Score and Prime MM-GBSA $\Delta G_{bind(NS)}$ for all the docking poses. The two selected representative poses are highlighted in light-gray

ligand	model	XP Glide Score	MM-GBSA $\Delta G_{bind(NS)}$	ligand	model	XP Glide Score	MM-GBSA $\Delta G_{bind(NS)}$
TCDD	AhR-4ghi	-7,9	-90,1	BNF	AhR-4ghi.2	-7,5	-83,0
	AhR-3h7w	-7,3	-88,8		AhR-4gs9	-8,3	-82,3
	AhR-3h7w	-7,3	-88,5		AhR-4zqd	-8,8	-79,9
	AhR-4xt2	-6,9	-85,9		AhR-3h7w	-8,7	-76,0
	AhR-4xt2	-6,9	-85,5		AhR-4xt2	-8,2	-74,3
	AhR-3f1o	-4,9	-85,3		AhR-4ghi	-7,1	-72,5
	AhR-3f1o	-4,9	-85,3		AhR-3f1o	-6,5	-69,4
TCDF	AhR-4gs9	-7,5	-93,4	FICZ	AhR-4ghi	-10,1	-74,7
	AhR-4xt2	-6,1	-91,4		AhR-3h7w	-8,1	-74,0
	AhR-4ghi	-8,5	-90,0		AhR-4xt2	-7,8	-70,2
	AhR-4zqd	-7,6	-86,5		AhR-4zqd	-7,5	-68,5
	AhR-3h7w	-7,1	-86,1	IR trans	AhR-4zqd	-7,0	-72,4
	AhR-3f1o	-6,2	-81,6		AhR-4gs9	-6,9	-71,7
PCB126	AhR-4ghi.2	-8,5	-104,1		AhR-3h7w	-8,0	-67,7
	AhR-4gs9	-7,4	-103,7		AhR-3f1o	-6,3	-67,5
	AhR-4ghi	-8,1	-99,0		AhR-4xt2	-7,5	-65,7
	AhR-4zqd	-7,5	-97,0		AhR-4ghi	-6,9	-60,7
	AhR-3h7w	-7,4	-95,8	LEFL	AhR-3f1n	-5,7	-79,3
	AhR-4xt2	-6,6	-92,6		AhR-4gs9	-6,4	-70,9
	AhR-3f1o	-4,7	-89,8		AhR-4ghi.2	-6,3	-69,5
BaP	AhR-4ghi.2	-7,4	-74,3		AhR-4ghi	-9,1	-68,1
	AhR-3h7w	-8,8	-71,9		AhR-4zp4	-4,9	-65,5
	AhR-4zqd	-7,6	-69,6		AhR-3h82	-8,1	-64,8
	AhR-4xt2	-6,5	-67,1		AhR-4zqd	-7,4	-63,1
3MC	AhR-4zqd	-8,3	-86,8		AhR-3h7w	-5,6	-59,9
	AhR-3h7w	-7,9	-83,5		AhR-4xt2	-5,4	-55,2
	AhR-4xt2	-7,5	-79,4		AhR-3f1o	-6,0	-53,2
DBA	AhR-4gs9	-8,6	-86,8				
	AhR-4zqd	-8,4	-84,1				
	AhR-4ghi	-8,3	-83,3				
	AhR-3h7w	-9,2	-82,2				
	AhR-4xt2	-8,8	-78,7				

Table S5. MM-GBSA ΔG_{bind} mean values in the last 8 ns of MD simulations for the selected docking poses. The pose with the lowest ΔG_{bind} value is highlighted in light gray

ligand	ΔG_{bind} mean [kcal·mol^{-1}]	sd [kcal·mol^{-1}]
TCDD-3h7w	-36.8	1.9
TCDD-4ghi	-34.8	1.8
TCDF-4ghi	-34.0	1.8
TCDF-4gs9	-32.7	1.9
PCB126-4ghi.2	-38.0	2.0
PCB126-4xt2	-36.1	1.9
BaP-4ghi.2	-34.7	2.3
BaP-3h7w	-30.0	2.1
3MC-4zqd	-38.3	3.0
3MC-4xt2	-31.0	2.3
DBA-4gs9	-37.6	2.6
DBA-4ghi	-34.1	1.9
BNF-3h7w	-38.7	2.0
BNF-4ghi.2	-37.0	2.4
FICZ-4ghi	-40.4	2.3
FICZ-4xt2	-35.9	3
IR trans-4zqd	-39.5	3.0
IR trans-4ghi	-34.0	4.0
LEFL-3f1n	-36.6	2.0
LEFL-4gs9	-31.0	2.0

Table S6. Relative ligand binding affinity of test chemicals for mutant AhRs. The relative affinity (IC_{50}) of each test chemical for the AhR ligand was determined from concentration-dependent inhibition curves obtained using [^{3}H]TCDD ligand binding analysis, as described under Materials and Methods. The mean IC_{50} ± standard deviation was determined using three-parameter non-linear regression with nine independent reactions.

AhR Mutant	AhR Ligand	IC_{50}[M]	SD [M]	-LogIC_{50}
P291L	TCDF	4.35E-11	2.20E-10	10.36
	BaP	6.04E-07	1.82E-07	6.22
M342A	3MC	2.48E-07	2.93E-08	6.61
	PCB126	9.33E-09	1.76E-07	8.03
	DBA	6.72E-09	1.13E-08	8.17
	BNF	9.60E-09	4.38E-09	8.02
	FICZ	2.07E-11	5.86E-11	10.68
	IR	1.26E-08	4.60E-09	7.90
	LEFL	9.03E-09	2.74E-08	8.04
M334A	3MC	3.47E-08	1.74E-08	7.46
	PCB126	7.41E-08	5.79E-09	7.13
	DBA	7.33E-09	1.56E-08	8.13
H331A	3MC	1.41E-10	3.83E-11	9.85
S330A	LEFL	6.29E-06	1.64E-06	5.20
S359A	BNF	4.71E-08	3.75E-10	7.33
	FICZ	4.10E-09	1.30E-09	8.39
	IR	9.20E-08	1.98E-09	7.04
	LEFL	non-converged	non-converged	non-converged
C327A	DBA	1.46E-08	3.15E-09	7.83
	BNF	4.05E-08	8.92E-09	7.39
	FICZ	4.32E-09	2.59E-08	8.36
	IR	7.58E-09	2.16E-08	8.12

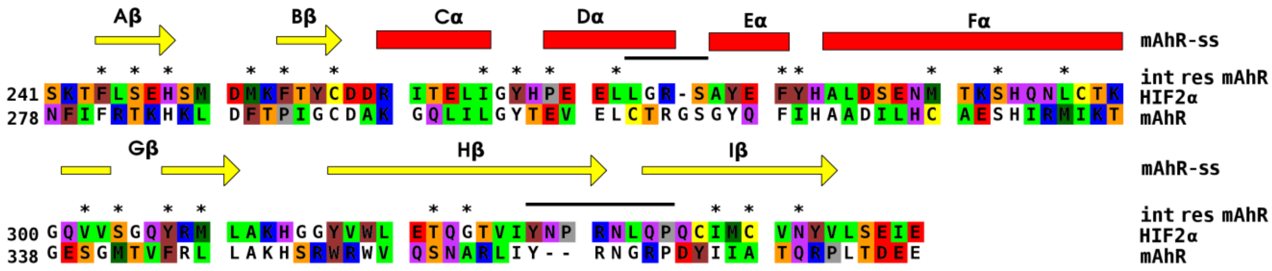

Figure S1. Sequence alignment between HIF-2α and mAhR used for Homology Modeling. Human HIF-2α sequence: Uniprot ID: Q99814, residues: 241-349; mAhR: Uniprot ID: P30561, residues 278-384). Amino acid residues are colored by physicochemical properties, secondary structures attributed by DSSPcont to the AhR-3h7w model are reported. Internal residues are marked by asterisks and residues subjected to loop-modeling refinement (Dα-Eα: 310-314 and Hβ-Iβ: 365-370) are underlined.

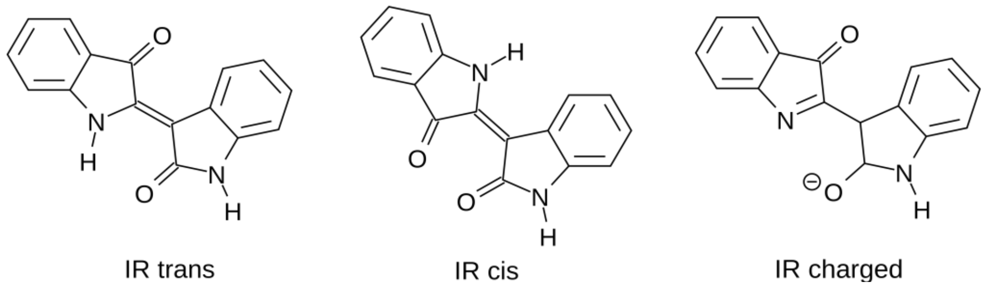

Figure S2. The three possible forms of IR at pH = 7 found by Epik.

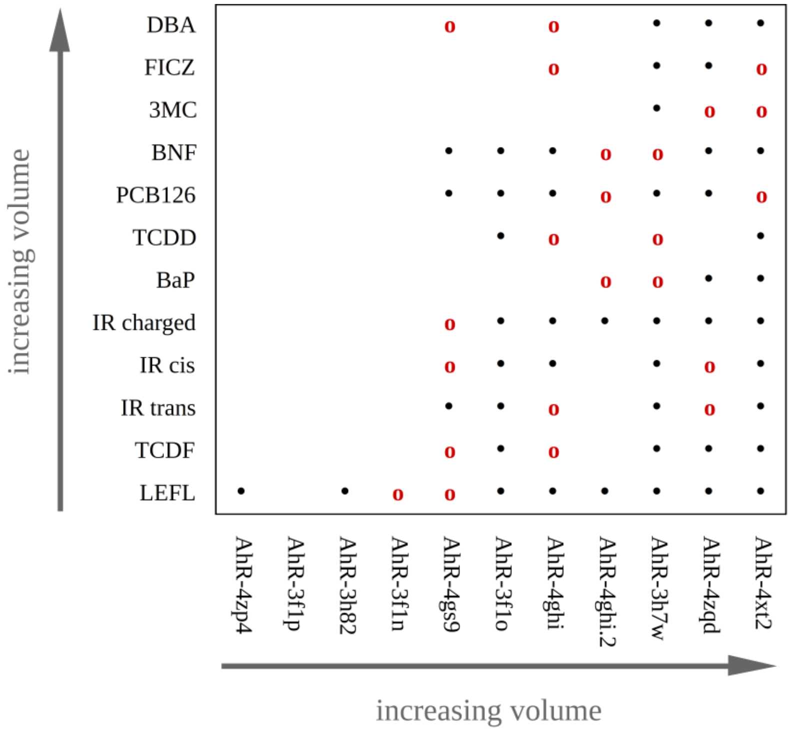

Figure S3. Docking matrix; each symbol indicates that a pose was found for that ligand in that model. Red circles stand for the two poses chosen as representative of the binding mode variability of each ligand. Ligands and cavities are ordered by increasing occupancy or volume, respectively.

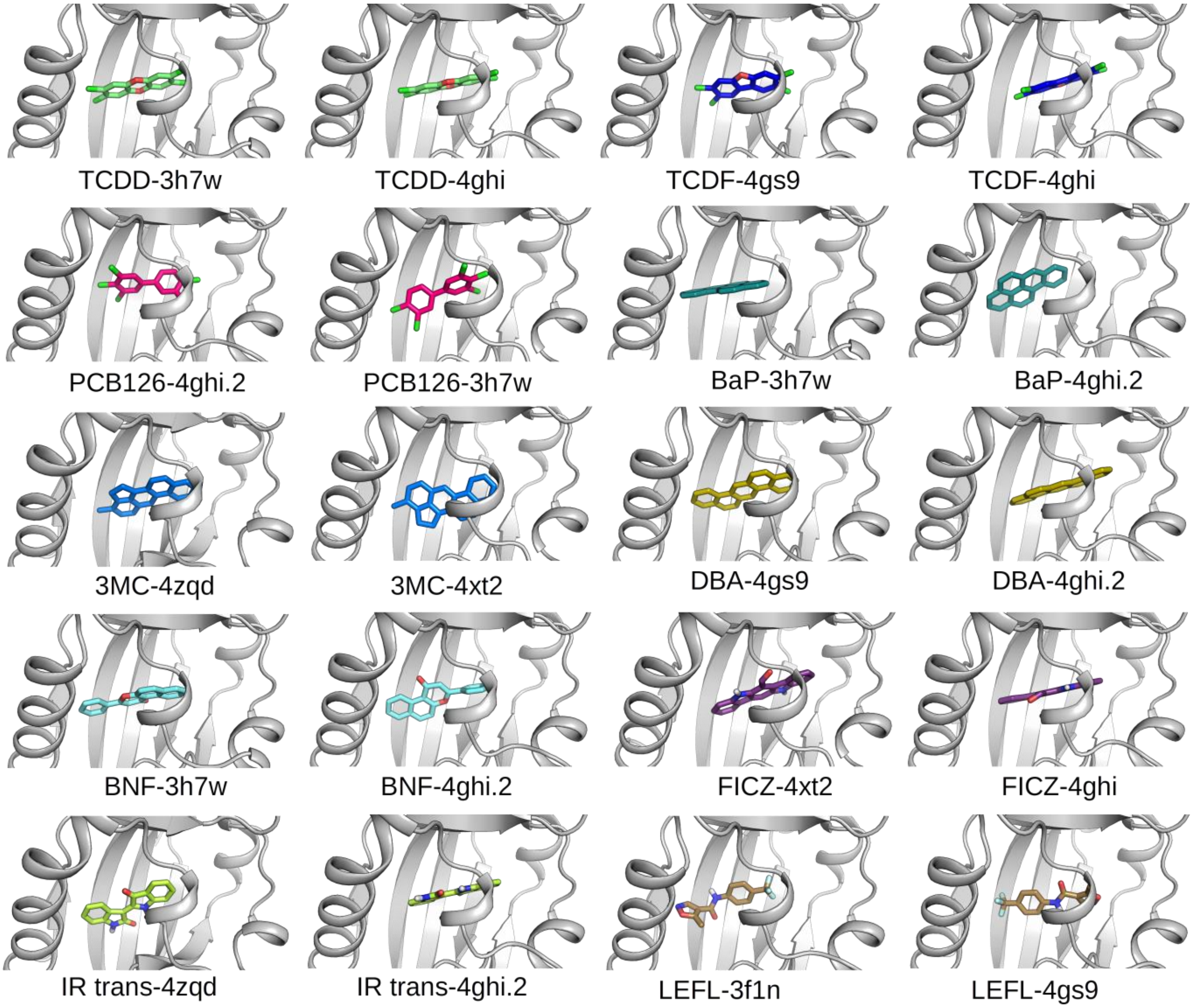

Figure S4. Three-dimensional representation of the two representative docking poses selected for each ligand. Protein is shown as cartoons, ligands as sticks.

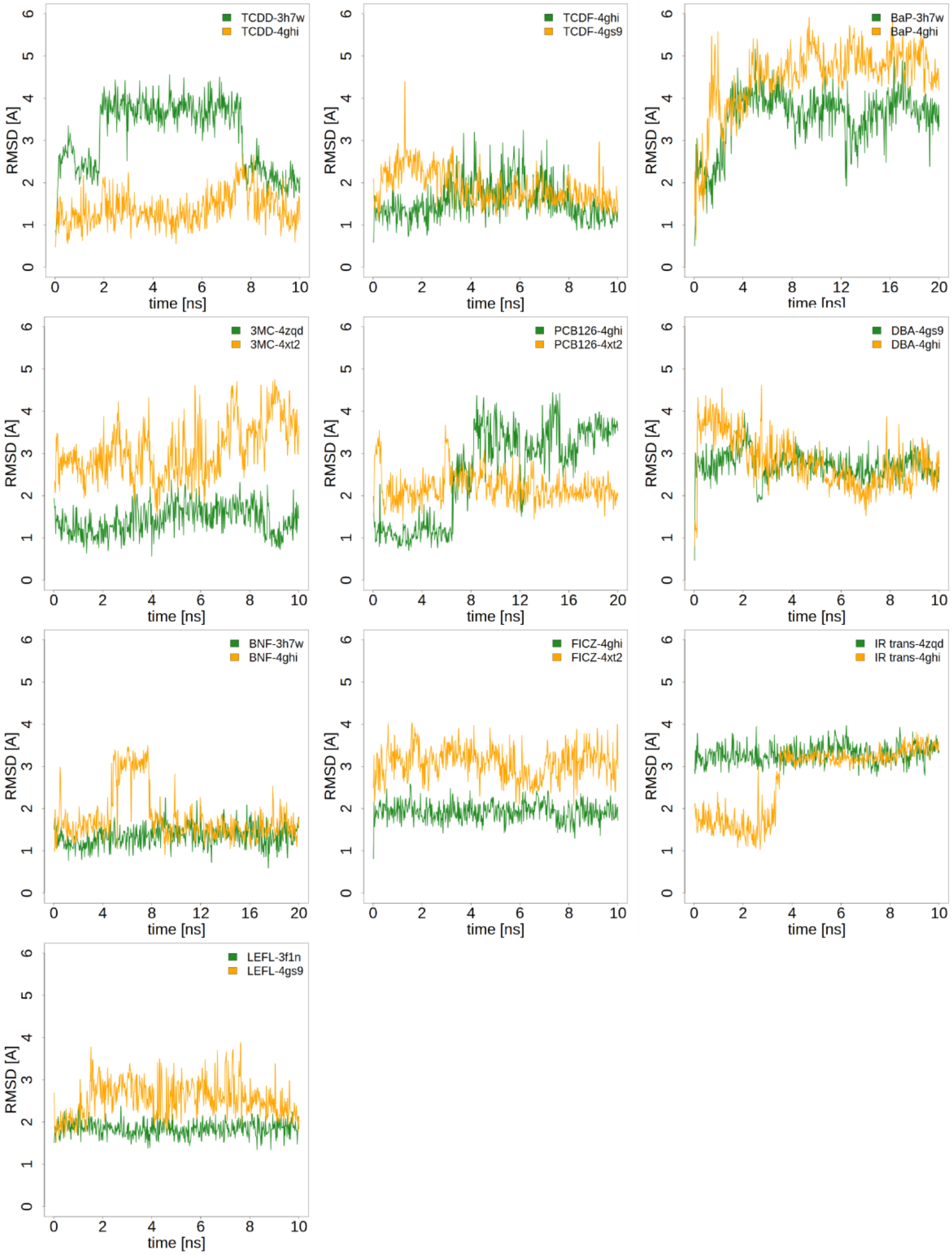

Figure S5. RMSD graphs in the MD simulations of the two representative poses of each ligand (the pose with the lowest ΔG_{bind} is shown in green). RMSD is calculated against the initial docking pose.

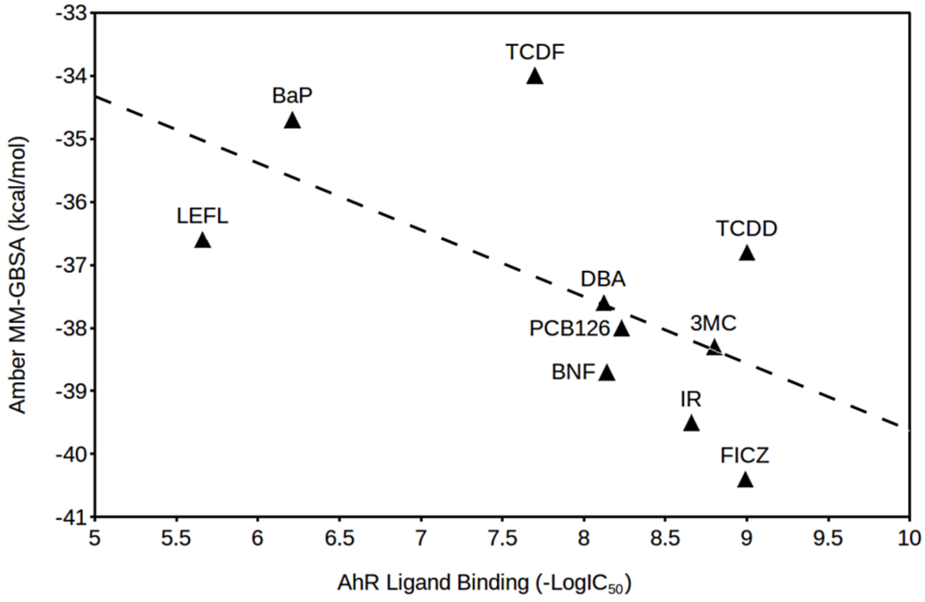

Figure S6. Correlation between the Amber MM-GBSA ΔG_{bind} and the experimental binding affinity of each compound ($-LogIC_{50}$). Pearson correlation coefficient $R^2 = 0.37$; Spearman rank-order correlation $\rho=0.64$ with 2-tail p-value=0.048.

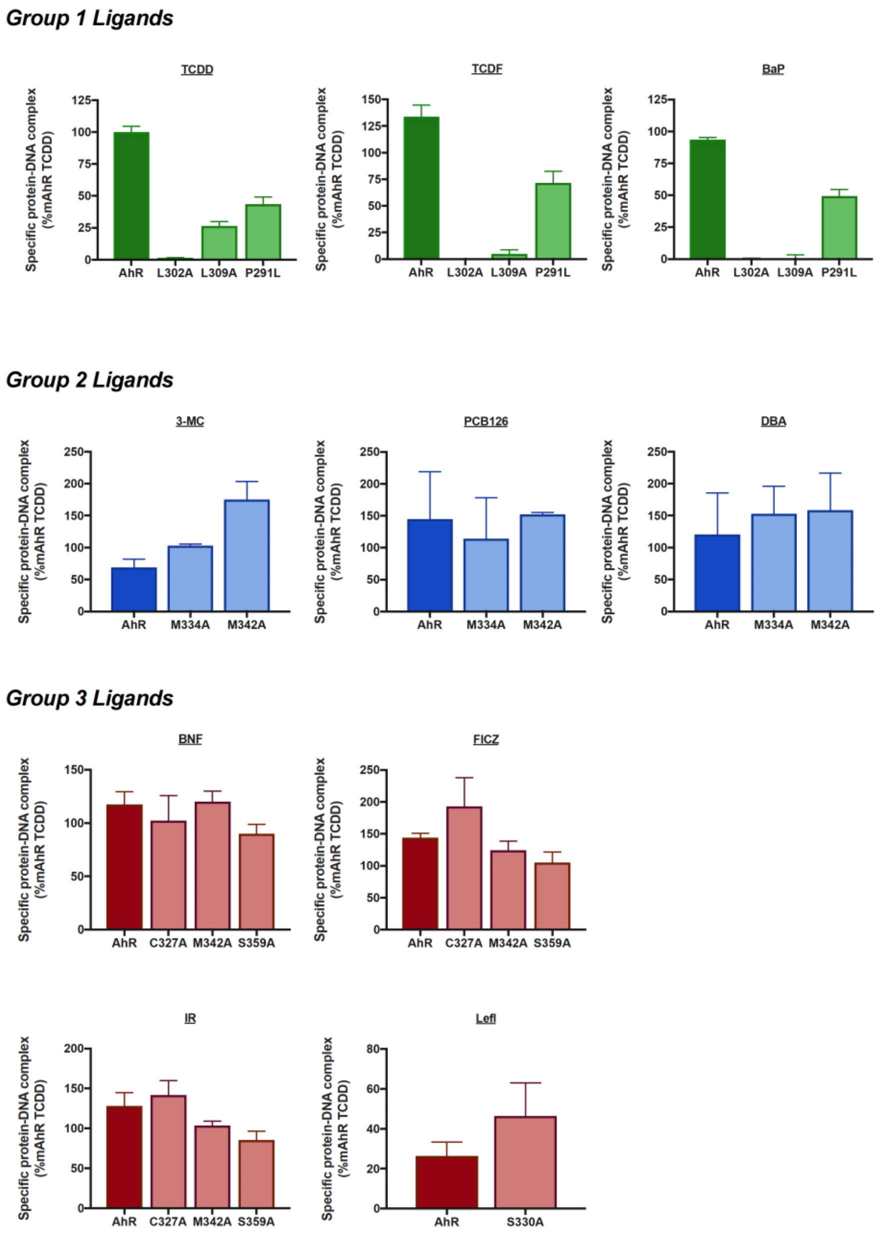

Figure S7. Relative ability of test chemicals to stimulate AhR transformation/DNA binding of wild-type and mutant AhRs. Single concentrations of AhR ligands were used to assess AhR transformation/DNA binding measured as specific protein-DNA complex (% mAhR TCDD) by Gel Retardation Analysis as described in the Material and Methods section. Maximal concentrations of AhR ligands were used and include BNF (10 µM), BaP (10µM), 3MC (10 µM), IR (1 µM), DBA (10 µM), FICZ (1 µM), PCB126 (100 nM), LEFL (100 µM), TCDD (20 nM), and TCDF (100 nM). Values represent the Three-parameter non-linear regression performed with nine independent reactions for mean specific protein-DNA complex (%mAhR TCDD) ± standard deviation of two independent reactions.
